# Role of sirtuins in epigenetic regulation and aging control

**DOI:** 10.18699/vjgb-24-26

**Published:** 2024-04

**Authors:** E.M. Samoilova, S.E. Romanov, D.A. Chudakova, P.P. Laktionov

**Affiliations:** Novosibirsk State University, Novosibirsk, Russia Engelhardt Institute of Molecular Biology of the Russian Academy of Sciences, Moscow, Russia; Novosibirsk State University, Novosibirsk, Russia Institute of Molecular and Cellular Biology of the Siberian Branch of the Russian Academy of Sciences, Novosibirsk, Russia; Federal Center of Brain Research and Neurotechnologies of the Federal Medical Biological Agency of Russia, Moscow, Russia; Novosibirsk State University, Novosibirsk, Russia Institute of Molecular and Cellular Biology of the Siberian Branch of the Russian Academy of Sciences, Novosibirsk, Russia

**Keywords:** sirtuins, aging, protein deacetylation, epigenetic regulation, сиртуины, старение, деацетилирование белков, эпигенетическая регуляция

## Abstract

Advances in modern healthcare in developed countries make it possible to extend the human lifespan, which is why maintaining active longevity is becoming increasingly important. After the sirtuin (SIRT) protein family was discovered, it started to be considered as a significant regulator of the physiological processes associated with aging. SIRT has deacetylase, deacylase, and ADP-ribosyltransferase activity and modifies a variety of protein substrates, including chromatin components and regulatory proteins. This multifactorial regulatory system affects many processes: cellular metabolism, mitochondrial functions, epigenetic regulation, DNA repair and more. As is expected, the activity of sirtuin proteins affects the manifestation of classic signs of aging in the body, such as cellular senescence, metabolic disorders, mitochondrial dysfunction, genomic instability, and the disruption of epigenetic regulation. Changes in the SIRT activity in human cells can also be considered a marker of aging and are involved in the genesis of various age-dependent disorders. Additionally, experimental data obtained in animal models, as well as data from population genomic studies, suggest a SIRT effect on life expectancy. At the same time, the diversity of sirtuin functions and biochemical substrates makes it extremely complicated to identify cause-and-effect relationships and the direct role of SIRT in controlling the functional state of the body. However, the SIRT influence on the epigenetic regulation of gene expression during the aging process and the development of disorders is one of the most important aspects of maintaining the homeostasis of organs and tissues. The presented review centers on the diversity of SIRT in humans and model animals. In addition to a brief description of the main SIRT enzymatic and biological activity, the review discusses its role in the epigenetic regulation of chromatin structure, including the context of the development of genome instability associated with aging. Studies on the functional connection between SIRT and longevity, as well as its effect on pathological processes associated with aging, such as chronic inflammation, fibrosis, and neuroinflammation, have been critically analyzed

## Abstract (ru)

Достижения современного здравоохранения в развитых странах позволили увеличить продолжительность жизни, из-за чего все более актуальным становится сохранение активного долголетия. С момента открытия белки семейства сиртуинов рассматривались в качестве значимых регуляторов физиологических процессов, ассоциированных со старением. Сиртуины проявляют деацетилазную, деацилазную, АДФ-рибозилтрансферазную активность и модифицируют множество белковых субстратов, включая компоненты хроматина и регуляторные белки. Столь многофакторная система регуляции затрагивает ряд процессов, таких как клеточный метаболизм, функции митохондрий, эпигенетическую регуляцию, репарацию ДНК и прочие. Неудивительно, что активность белков-сиртуинов затрагивает проявление классических признаков старения организма: клеточное старение, нарушения метаболизма, митохондриальную дисфункцию, геномную нестабильность и нарушение эпигенетической регуляции. Непосредственное изменение активности сиртуинов в клетках человека также рассматривается в качестве маркера старения и вовлечено в генез различных возраст-зависимых патологических состояний. Кроме того, экспериментальные данные, полученные на модельных животных, а также результаты популяционных геномных исследований позволяют предположить влияние сиртуинов на продолжительность жизни. Вместе с тем многообразие функций сиртуинов и биохимических субстратов делает крайне нетривиальным выявление причинно-следственных связей и непосредственной роли сиртуинов в контроле функционального состояния организма. Однако влияние сиртуинов на эпигенетическую регуляцию экспрессии генов в ходе старения и при патологиях – один из наиболее важных аспектов поддержания гомеостаза органов и тканей. Представленный обзор посвящен разнообразию белков- сиртуинов у человека и модельных животных. Помимо краткого описания основных ферментативных и биологических активностей сиртуинов, рассматривается роль сиртуинов в эпигенетической регуляции структуры хроматина, в том числе в контексте развития нестабильности генома, ассоциированной со старением. Проведен критический анализ работ по исследованию функциональной связи сиртуинов и долголетия, а также влияния сиртуинов на ассоциированные со старением патологические процессы, такие как хроническое воспаление, фиброз и нейровоспаление.

## Introduction

The first representative of sirtuin proteins, Sir2 (silent information regulator 2), was discovered in the budding yeast Saccharomyces cerevisiae. It was initially described as a key transcription repressor at HM loci responsible for the matingtype switching of yeast (Ivy et al., 1986). Subsequently, it was confirmed that Sir2 is needed to suppress the expression of transgenes near telomeres and the silencing of retrotransposons that have been integrated into tandem repeats of ribosomal DNA (Gottschling et al., 1990; Bryk et al., 1997). Its main function is the NAD+-dependent histone deacetylation (Imai et al., 2000; Smith et al., 2000). Deletion of the Sir2 gene was found to approximately halve the lifespan of S. cerevisiae. Conversely, the overexpression of Sir2 increases it by a third (Sinclair, Guarente, 1997; Kaeberlein et al., 1999). Sir2 homologues, united by the name “sirtuins” (silent information regulator two proteins), were found in all domains of living organisms from bacteria and archaea to humans (Frye, 2000). Therefore, a direct relationship between Sir2 activity and yeast lifespan initiated research into the role of the NADdependent Sir2 family deacetylases in the regulation of the aging processes. Seven sirtuins (SIRT1–SIRT7) were found in mammals, where SIRT1 has the greatest homology to yeast Sir2 (Frye, 2000).

**Fig. 1.**
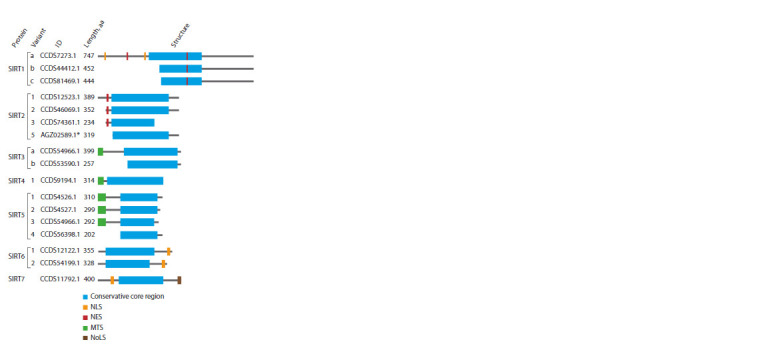
Human sirtuins diversity. Information on protein isoforms annotated in the CCDS database is provided (Pruitt et al., 2009). Functional protein regions are marked with colored rectangles. * Amino acid sequence identifier from the NCBI Proteins Database (Sayers et al., 2022).

Sirtuins of higher eukaryotes were grouped into class III HDAC due to their specific function and structural features (Gray, Ekström, 2001). All human sirtuins are characterized by a common conserved core region of 250–270 amino acids in length (Fig. 1). This protein fragment consists of a Rossman fold domain, which is characteristic of many NAD+-dependent proteins, and a small domain, which consists of a Zn-binding and a helical modules (Moniot et al., 2013).

The main differences between homologues are found in the N- and C-terminal domains, which can contain signals for nuclear or nucleolar localization (NLS and NoLS, respectively), nuclear export (NES) or mitochondrial transport (MTS) in different proteins (see Fig. 1). It is worth noting that the seven human sirtuin genes encode at least 23 protein isoforms (Rack et al., 2014; Zhang X. et al., 2021). Features of minor isoforms can be either an absence of transport signal sequences or an altered core structure, due to which they can have original functions (Rack et al., 2014; Du Y. et al., 2018).

Sirtuins are involved in the regulation of different intracellular processes: cellular metabolism, mitochondrial functions, chromatin remodeling, and response to oxidative stress (Wu et al., 2022). At the body level, sirtuins affect metabolism, aging, and carcinogenesis. Changes in the activity of sirtuins in human cells and model organisms are considered to be markers of aging (Kumar et al., 2014; Zhang J. et al., 2016), as well as factors affecting overall life expectancy (Roichman et al., 2021). Within the frames of the presented review, the regulatory activities of sirtuins, their participation in epigenetic regulation and involvement in the genesis of age-related diseases are analyzed

## Sirtuins biochemical activity

**Fig. 2.**
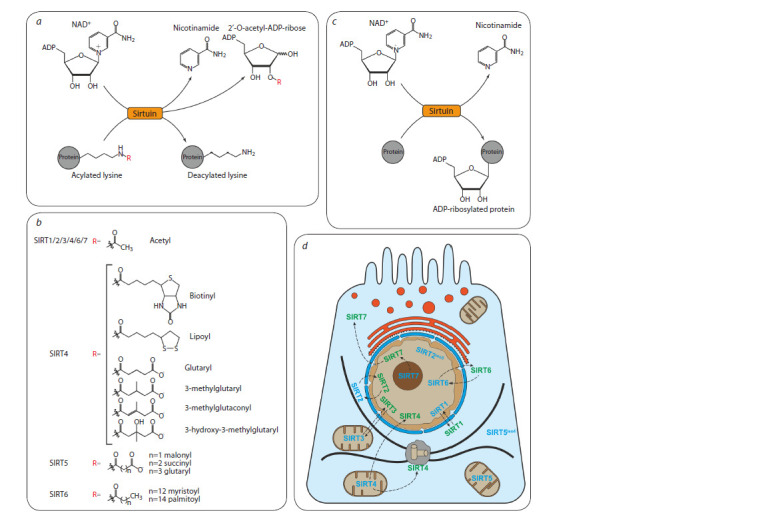
Human sirtuins molecular function а – deacylase activity of sirtuins; b – some functional groups that can be removed by different sirtuins; c – ADP-ribosyltransferase reaction involving sirtuins; d – localization of human sirtuins in cells. The protein is marked with blue text if it is placed in the area of constitutive localization in the diagram, or green otherwise

First of all, sirtuins are known as enzymes-deacetylases of histones and non-histone proteins (Fig. 2, a) (Sauve et al., 2006). In addition, sirtuins are able to remove different acyl residues (see Fig. 2, a, b). For example, SIRT5 predominantly deacylates lysine residues which are modified by succinyl, malonyl or glutaryl groups (Du J. et al., 2011; Tan et al., 2014). Some sirtuins combine deacetylase and deacylase activity. For instance, SIRT6 can remove residues of myristic and palmitic fatty acids (Jiang et al., 2013; Zhang X. et al., 2017), and SIRT4 can remove residues of lipoic acid, biotin, glutarate and its derivatives (Laurent et al., 2013; Mathias et al., 2014).

Sirtuins are also able to perform mono-ADP-ribosylation of proteins (Frye, 1999). In such cases, sirtuins transfer ADP-ribose from NAD+ directly to the amino acid residues of arginine, yielding nicotinamide (see Fig. 2, c) (Fahie et al., 2009). ADP-ribosyltransferase activity in mammals has now been described for SIRT4, SIRT6, and SIRT7. However, the total number of their targets is small. For example, SIRT4 ADPribosylates glutamate dehydrogenase (GDH) in pancreatic beta cells and inhibits its activity by limiting glutamate and glutamine metabolism (Haigis et al., 2006). Under oxidative stress, SIRT6 is recruited to DNA breakpoints and induces damage repair by ADP-ribosylation of poly-ADP-ribosyltransferase PARP1, one of the most important regulators of DNA repair (Mao et al., 2011). Moreover, SIRT6 is recruited in fibroblasts to the 5′ untranslated region of LINE1 retrotransposon and suppresses its expression by ADP-ribosylation of transcriptional repressor KAP1 (Van Meter et al., 2014). Auto-ADPribosylation of SIRT7 in humans regulates its binding to genes enriched with mH2A1.1 histone modification, which is important for glucose homeostasis (Simonet et al., 2020).

## Sirtuin function and modulation in cells

Sirtuins are divided into predominantly nuclear (SIRT1/6/7), cytoplasmic (SIRT2), and mitochondrial (SIRT3/4/5) (Michishita et al., 2005). However, their intracellular localization can change both during the cell cycle and under various stimuli (see Fig. 2, d ).

SIRT1 is most often found in the nucleus, where it regulates the structure of chromatin and the activity of many regulatory proteins. It has also been found in the cytoplasm (Bai, Zhang, 2016). SIRT1 is assumed to be present in the nucleus, but under the influence of unknown stimuli it can be transported to the cytoplasm due to a nuclear export signal (NES) (Sun, Fang, 2016). The transfer of SIRT1 into cytoplasm is also observed during cellular aging, accompanied by autophagocytosis of SIRT1 in lysosomes (Xu et al., 2020; Wang L. et al., 2021).

SIRT6 is more commonly found in the nucleus, but it can be observed in the cytoplasm of liver cells in response to increased levels of saturated fatty acids. In this case, it deacetylates and activates acyl-CoA synthetase 5 (ACSL5), one of the fatty acid oxidation enzymes (Hou et al., 2022). In mouse macrophages, the SIRT6 fraction is constantly present in the cytoplasm and stimulates the secretion of TNF-α protein by removing its meristyl modification (Bresque et al., 2022).

SIRT7 is the only sirtuin that is enriched in the nucleoli, where it is involved in the transcription of ribosomal genes (Ford et al., 2006; Kiran et al., 2013). Under stress, when rRNA production is disrupted, SIRT7 moves to the nucleoplasm or cytoplasm (Chen et al., 2013; Zhang P.-Y. et al., 2016), where it interacts with a variety of proteins (Tsai et al., 2012; Lee et al., 2014). Although the exact function of cytoplasmic SIRT7 remains unknown, it is presumably associated with the regulation of replicative senescence (Kiran et al., 2013).

SIRT2 is more often found in the cytoplasm. It is involved in the regulation of cell cycle, fatty acids and carbohydrates metabolism, oxidative stress response and many other processes. In the interphase, it localizes to microtubules and deacetylates α-tubulin (North et al., 2003). During the G2/M transition, SIRT2 temporarily migrates to the nuclei and deacetylates histone H4 by lysine 16, thereby modulating chromatin condensation (Vaquero et al., 2006). SIRT2 passes from the nucleus to the cytoplasm due to the nuclear export signal (NES) at the N-terminus (North, Verdin, 2007). Its nuclear export depends on posttranslational modifications and can be disrupted by various stimuli (North, Verdin, 2007). For example, infection of HeLa cells with Listeria monocytogenes bacterium leads to dephosphorylation of SIRT2 by S25 residue, which leads to an increase in the nuclear concentration of the protein. Here, it mediates the repression of immune response genes by deacetylation of H3K18ac (Eskandarian et al., 2013; Pereira et al., 2018).

The transfer of SIRT2 to the nucleus is also observed in glioblastoma cells and other types of tumors, and patients with higher SIRT2 in the nuclei of tumor cells have a worse prognosis for glioma (Imaoka et al., 2012; Eldridge et al., 2020). Reduction in nuclear exports can also be achieved through alternative splicing. For example, the recently discovered SIRT2iso5 isoform with unknown enzymatic activity does not have a nuclear export signal and is constitutively present in the nucleus, where it interacts with histone methyltransferases, and also suppresses transcription and replication of the hepatitis B virus (HBV) (Rack et al., 2014; Piracha et al., 2020).

SIRT3, SIRT4, and SIRT5 are predominantly found in the mitochondrial matrix and play a key role in cellular processes such as oxidative stress response, dissimilation, and apoptosis (Michishita et al., 2005). At the same time, in mice, a disruption of the SIRT3 function leads to a significant increase in acetylation of mitochondrial proteins. Knockout of SIRT4 and SIRT5 has a significantly weaker effect (Lombard et al., 2007; Finkel et al., 2009). However, mitochondrial proteins are also found in other cellular compartments. SIRT3 is detected in the cell nucleus, where it is involved in the regulation of heterochromatin structure and NHEJ-dependent DNA repair (Sengupta, Haldar, 2018; Diao et al., 2021). SIRT4 interacts with the centrosome at the end of the G2 phase, and under mitochondrial stress moves to the nucleus, although the function of this remains unclear (Ramadani-Muja et al., 2019; Bergmann et al., 2020). In addition, only three out of four studied SIRT5 isoforms contain a signal of mitochondrial localization (see Fig. 1). At the same time, the shortest isoform, SIRT5iso4, does not have it, and is found in the cytoplasm of cells (Du Y. et al., 2018).

## Sirtuins in chromatin and epigenetic regulation

The key parts of epigenetic mechanisms of gene regulation are specific marks – histone modifications and DNA methylation – as well as effector proteins capable of establishing and recognizing such tags. The coordinated work of such a system determines the properties of chromatin, which, in its turn, determines the activity of genes and the stability of the genome. Sirtuins are involved both in the direct control of histone modifications and in the activity and stability of regulatory factors.

SIRT1 is involved in the formation of heterochromatin by removing H4K16Ac, H3K9Ac, and H1K26Ac marks, as well as interacting with Suv39h1 histone methyltransferase, which is responsible for the installation of a key histone modification of the H3K9me3 constitutive heterochromatin (Vaquero et al., 2004, 2007; Bosch-Presegué et al., 2011). Thus, SIRT1 participates in the establishment of H3K9me3 by removing H3K9Ac, as well as by direct interaction with Suv39h1, which increases the specific activity of the latter as a result of conformational changes, deacetylation of K266 in the catalytic SET domain, and also increases resistance to proteosomal degradation by suppressing ubiquitination at the K87 site in Suv39h1 chromodomain (Vaquero et al., 2007; Bosch-Presegué et al., 2011). Impaired SIRT1 function leads to a significant loss of HP1 and H3K9me3 in the composition of pericentromeric heterochromatin (Vaquero et al., 2007; Wang R.-H. et al., 2008; El Ramy et al., 2009).

It is important to note that such a significant effect on chromatin structure cannot but affect the expression of other regulatory factors. For example, the activation of SIRT1 has been found to increase proliferation, invasion, and accelerate epithelial-mesenchymal transition in pancreatic tumor cells, which is associated at least in part with suppression of gene expression of the FOXO3 and GRHL3 transcription factors (Leng et al., 2021).

SIRT1 is also involved in the regulation of DNA methylation, both at the level of transcription regulation and directly modulating the activity of DNA methyltransferases. Thus, in a mouse embryonic stem cell model, it was shown that SIRT1 deacetylates H1 and H4 histones in the promoter region of the Dnmt3l gene, suppressing its expression (Heo et al., 2017). A deficiency of SIRT1 leads to increased methylation of genomic DNA, as well as to deregulation of imprinted genes (Heo et al., 2017). It is interesting to note that the same study showed that SIRT1 is able to deacetylate the Dnmt3l protein, which reduces its stability (Heo et al., 2017). The effect of SIRT1 on human DNMT1 DNA methyltransferase has also been demonstrated. Moreover, deacetylation of lysine residues in the catalytic domain led to an increase in methyltransferase activity, and deacetylation in GK linker region led to its decrease (Peng et al., 2011). During differentiation of human macrophages, SIRT1 and SIRT2 physically interact with the DNMT3B DNA methyltransferase to prevent aberrant activation of pro-inflammatory genes (Li T. et al., 2020).

In addition to DNA methyltransferases, SIRT1 also affects the activity of many other non-histone targets. Thus, deacetylation of p53 protein under the action of SIRT1 led to the repression of apoptosis in H1299 cells (Luo et al., 2001; Vaziri et al., 2001). In addition, deacetylation of p53 leads to suppression of its regulatory activity as an oncosuppressor (Ong, Ramasamy, 2018).

Deacetylation of Ku70 protein, one of the key components of NHEJ DNA repair, by SIRT1 activates DNA repair (Jeong et al., 2007). These examples show that SIRT1 stimulates cell survival in case of DNA damage. However, SIRT1 is also able to deacetylate the p65 subunit of NF-κB, which on the contrary leads to activation of TNF-α-induced apoptosis in non-small cell lung cancer cells (Yeung et al., 2004).

Deacetylation of FOXO transcription factors (FOXO1, FOXO3, FOXO4) under the action of SIRT1 can lead to both activation of apoptosis (FOXO1) and cell cycle arrest and suppression of apoptosis (FOXO3) (Brunet et al., 2004; Yang et al., 2005). In turn, deacetylation of FOXO4 increases its protective effect under oxidative stress (van der Horst et al., 2004). In addition to the above, SIRT1 is involved in regulating the activity of transcription factors that control the response to hypoxia, metabolism, cell invasion, and proliferation.

SIRT3 is largely described as a regulator of mitochondrial functions. However, in nuclei, its role in NHEJ DNA repair due to the removal of H3K56ac histone modification has been shown (Sengupta, Haldar, 2018). SIRT3 also plays a role in deacetylation of H3 histone at lysine residue 27, which is associated with repression of transcription of the FOS transcription factor gene and prevention of TNF-α-induced inflammatory and profibrotic responses in rat cardiomyocytes (Palomer et al., 2020).

At the chromatin level in HEK293T cells, SIRT3 has been shown to be able to directly interact with nuclear lamina components LaminB1 and LBR, as well as the HP1α, HP1γ, and KAP1 heterochromatin proteins (Diao et al., 2021). Moreover, deletion of SIRT3 in human mesenchymal stem cells (MSCs) resulted in dissociation of lamina-associated domains and a decrease in the abundance of the H3K9me3, HP1α, and KAP1 heterochromatin protein markers, as well as the LaminB1 nuclear membrane component (Diao et al., 2021). Restoring SIRT3 had the opposite effect (Diao et al., 2021). At the same time, it is important to note that deletion of SIRT3 led to accelerated cellular senescence, and all the detected effects are (among other things) its classical manifestations. In this regard, despite the obviousness of the phenotype, as well as the participation of SIRT3 in the regulation of other processes associated with cellular aging, the cause-and-effect relationship may be more complex

SIRT6 is also involved in epigenetic regulation. One of its first discovered activities at the level of chromatin regulation was the ability to deacetylate the H3 histone by K9 and K56 (Kawahara et al., 2009). SIRT6 acts as a co-repressor of such transcription factors as NF-κB and HIF-1α by regulating the H3K9 acetylation (Kawahara et al., 2009; Zhong et al., 2010). Deacetylation of H3K9 under the action of SIRT6 is involved in the regulation of telomeres and gene expression (Michishita et al., 2008; Zhong et al., 2010). For example, the control of mouse embryonic stem cell differentiation depends on the removal of the H3K56ac and H3K9ac marks in the promoter regions of the Oct4, Sox2, and Nanog genes (Etchegaray et al., 2015). In addition, SIRT6 is able to directly deacetylate DNMT1 DNA methyltransferase, which reduces its stability (Jia et al., 2021; Subramani et al., 2023).

Increased expression of SIRT6 leads to destabilization of DNMT1 and a decrease in methylation of the NOTCH1 and NOTCH2 gene promoters, which leads to a predominant osteogenic differentiation of the MSCs from adipose tissue (Jia et al., 2021). In non-small cell lung cancer cell cultures, decreased SIRT6 expression leads to stabilization of DNMT1 and methylation of the promoter of the NOTCH1 gene, which is involved in oncogenesis and metastasis (Subramani et al., 2023). As mentioned earlier, SIRT6, through ADP-ribosylation of the KAP1 transcription repressor, is involved in suppressing LINE1 expression and maintaining genome stability (Van Meter et al., 2014).

SIRT7 is the only sirtuin localized in the nucleolus, where it plays a key role in the formation of transcriptionally inactive heterochromatin by recruiting DNMT1, SIRT1, and SMARCA5 to ribosomal DNA repeats (Ianni et al., 2017; Paredes et al., 2018). Compact state of chromatin is needed to prevent homologous recombination between repetitive rDNA sequences. Therefore, a disruption of the SIRT7 function leads to the formation of active chromatin, instability of the rDNA region and genome, and accelerated cellular senescence (Ianni et al., 2017; Paredes et al., 2018). In addition, SIRT7 is involved in the regulation of R-loop – RNA-DNA complexes, which are also a potential factor in genome instability (Aguilera, García-Muse, 2012; Song et al., 2017).

It is interesting to note that in the drosophila model, it was shown that the area of distribution of R-loops increases with age, while their number remains unchanged (Hall, 2023). Defects in the processing of R-loops can lead to accumulation of DNA/RNA hybrids, single-stranded DNA fragments in the cytoplasm, which stimulates the immune response, chronic inflammation, apoptosis and senescence (Chatzidoukaki et al., 2021; Crossley et al., 2023). SIRT7 deacetylates and activates the DDX21 helicase involved in R-loop resolving (Song et al., 2017)

In addition, the role of SIRT7 in the deacetylation of the H3K18ac histone modification, which is needed to activate the repair of double-strand breaks (DSBs) in DNA, has been shown (Barber et al., 2012; Lin et al., 2016b; Vazquez et al., 2016). Direct interaction with the KAP1, HP1α, and HP1γ heterochromatin proteins, as well as components of the LBR and LaminB1 nuclear lamina in the HEK293T cells was also demonstrated for SIRT7 (Bi et al., 2020). Reduced SIRT7 in human MSCs led to accelerated senescence, heterochromatin destabilization, awakening of repeated sequences, and the cGAS-STING proinflammatory signaling pathway activation (Bi et al., 2020).

## Sirtuins and longevity

For the first time, the possible influence of sirtuins on longevity was discovered in trials with an overexpression of Sir2, which led to an increase in the number of budding cycles in the S. cerevisiae yeast (Kaeberlein et al., 1999). In studies of the Sir2 homologues, with SIR-2.1 protein in the Caenorhabditis elegans nematode and dSirt1 in Drosophila melanogaster, an increased lifespan was observed with their overexpression (Tissenbaum, Guarente, 2001; Rogina, Helfand, 2004). At the same time, an overexpression of Sir2 in yeast increases the replicative potential, but does not regulate the lifespan of quiescent cells, which is a key parameter in the chronological aging model of S. cerevisiae (Fabrizio et al., 2005). In nematodes, the positive effect of sirtuins depends on the genetic background and is not detected in some laboratories (Burnett et al., 2011; Viswanathan, Guarente, 2011; Schmeisser et al., 2013; Zhao et al., 2019).

In drosophila, the effect of dSirt1 overexpression on longevity is dose-dependent. At the same time, exceeding a certain expression threshold may shorten the lifespan due to its toxicity to certain organs (Griswold et al., 2008; Burnett et al., 2011; Whitaker et al., 2013). In particular, it has been demonstrated that induced tissue-specific overexpression of dSirt1 increases the median lifespan of flies only when the transgene is activated in adipose tissue, but not in muscles (Banerjee et al., 2012). Similarly, a 9–16 % increase in mouse lifespan was achieved by triggering transgenic SIRT1 specifically in hypothalamic cells (Satoh et al., 2013), whereas in an earlier study, an overexpression of SIRT1 in mice did not affect longevity, although it reduced the likelihood of cancer (Herranz et al., 2010).

The effect of overexpression of sirtuins on longevity has also been shown for dSirt4 and dSirt6, the drosophila sirtuins (Wood et al., 2018; Taylor et al., 2022). In a recent study, slowed aging and increased maximum lifespan under the influence of SIRT6 were shown in mice (Roichman et al., 2021). The effect of SIRT6 on longevity is associated with its participation in DNA repair (Tian et al., 2019). Moreover, the activity of species-specific SIRT6 variants in the context of repair correlates with the maximum lifespan of different rodent species (Tian et al., 2019).

Conflicting data have been obtained for SIRT7. In particular, it has recently been demonstrated that male mice with SIRT7 knockout have an increased median lifespan and exhibit a slower decrease in physiological parameters (Mizumoto et al., 2022). This result contrasts with observations from earlier studies, in which the SIRT7 knockout significantly shortened the lifespan. It is important to note that none of the experiments evaluating the effect of sirtuins on organismal longevity showed an extreme increase in the lifespan, and along with the difficulty in selecting correct experimental controls – as genetically close as possible – this leads to ambiguous conclusions about the role of sirtuins as autonomous factors of longevity (Brenner, 2022).

Data on the possible relationship between sirtuins and human life expectancy are, to some extent, confirmed by population genetics data. For example, the research of Dutch centenarians showed that carriers of the rs12778366 single nucleotide polymorphism have better glucose tolerance and a reduced risk of death (Figarska et al., 2013). The research on the Americans of European descent and populations of Georgia and Louisiana demonstrated the association of the rs7896005 polymorphism of the SIRT1 gene with longevity and telomere length in lymphocytes (Kim et al., 2012). In addition, SIRT1 rs3758391 and rs4746720 polymorphisms were associated with healthy aging in the Han Chinese (Zhang W.- G. et al., 2010). However, in a similar research of another population of Chinese centenarians, the relationship of these loci with life expectancy was not revealed (Lin et al., 2016a). A number of other studies have not revealed the association of genetic variants of the SIRT1 gene with longevity (Flachsbart et al., 2006; Willcox et al., 2008; Soerensen et al., 2013).

In the fifth intron of the SIRT3 gene, variability in the number of tandem repeats (VNTR) was identified, some variants of which acquire the properties of an allele-specific enhancer. The allele without this enhancer activity was practically not found among men over 90 years old, while no such correlation was observed in women (Bellizzi et al., 2005). Polymorphisms of the SIRT3 gene, rs11555236 and rs4980329, were associated with the life expectancy of women in an Italian population (Albani et al., 2014).

The rs107251 nucleotide polymorphism of the SIRT6 gene was associated with a more than five-year increase in life expectancy in the elderly in the United States, and the rs117385980 polymorphism was associated with longevity in Finns (TenNapel et al., 2014; Hirvonen et al., 2017). The SIRT6 allele with N308K/A313S substitutions, which has strong ADP-ribosyltransferase activity, was enriched in a group of centenarians among Ashkenazi Jews (Simon et al., 2022).

## Sirtuins in chronic inflammation

In addition to some evidence of a possible functional relationship with longevity, sirtuins have been shown to play a significant role in the development of age-associated diseases, in particular those arising from chronic inflammation. The SIRT1, SIRT2, and SIRT6 proteins counteract the inflammatory response by suppressing the NF-κB signaling pathway (Vazquez et al., 2021). The key element of this pathway – the transcription factor NF-κB, which controls the expression of immune response genes, consists of five subunits: p50, p52, p65 (RelA), RelB, and c-Rel (Vazquez et al., 2021).

There are several mechanisms that sirtuins can use to suppress the activity of the NF-κB signaling pathway. SIRT1 and SIRT2 are able to deacetylate the p65 subunit at lysine 310, directly inhibiting the activity of NF-κB. They can also prevent methylation of neighboring lysine residues (K314 and K315), which contribute to ubiquitination and degradation of p65 (Rothgiesser et al., 2010). SIRT6 interacts with p65 and deacetylates H3K9 in promoters of NF-κB target genes, thereby reducing inflammation (Kawahara et al., 2009). SIRT1 is also able to deacetylate and inhibit the activity of NF-κB transcriptional coactivators, such as PARP-1 and p300 histone acetyl transferase (Rajamohan et al., 2009).

Sirtuins also influence the TGF-β signaling pathway – which plays a key role in tubulointerstitial renal fibrosis – by stimulating the production of connective tissue growth factor (CTGF) (Isaka, 2018). Overexpression of SIRT1 suppresses TGF-β1-induced cellular apoptosis and fibrosis, and reduces CTGF expression by stimulating TGF-β1 in the kidneys of mice with unilateral ureteral obstruction (Ren et al., 2015). SIRT1 is also able to weaken TGF-β-dependent signaling by deacetylating SMAD3 and SMAD4 molecules, which inhibits the production of collagen, fibronectin, and MMP7 metalloprotease (Zhang Y. et al., 2017).

The effect of sirtuins on the Smad transcription factors is also important in cardiac fibrosis. This is because systematic knockout of the mouse SIRT6 gene disrupts inhibition of the TGF-β/Smad3 signaling pathway, the cause of cardiac fibrosis (Maity et al., 2020). In addition, SIRT1 produces a cardioprotective effect by deacetylating SMAD2/3 and reducing the activity of the TGF-β signaling pathway in mouse cardiac fibroblasts (Bugyei-Twum et al., 2018).

The SIRT3 protein has antifibrotic properties that weaken TGF-β-dependent signaling, and the suppression of SIRT3 activity can lead to the transformation of mouse and human cardiac fibroblasts into myofibroblasts – cells capable of producing extracellular matrix (Sundaresan et al., 2016). As expected, activators of sirtuins counteract fibrosis. Thus, honokiol – a SIRT3 activator – counteracts kidney fibrosis in mice with unilateral ureteral obstruction (Quan et al., 2020). Similarly, activation of both SIRT1 and SIRT3 by resveratrol attenuates cardiac fibrosis in mice by inhibiting the TGF-β/ Smad3 pathway (Liu et al., 2019).

The physiological effect of sirtuins in inflammation is also directly related to the effect on immune cells. For example, SIRT1 is involved in the transmission of inflammatory signals in mouse dendritic cells by modulating the balance of type 1 pro-inflammatory T helper cells and Foxp3(+) anti-inflammatory regulatory T cells. A deficiency of SIRT6 in macrophages leads to inflammation with increased acetylation and greater stability of FoxO1 (Woo et al., 2016, 2018). SIRT4 also has an anti-inflammatory effect, since its deficiency can increase inflammation and promote macrophage infiltration and the development of cellular hepatocarcinoma in humans (Li Z. et al., 2019). In mouse liver cells, SIRT3 inhibits the production of pro-inflammatory chemokines and some profibrotic factors (LoBianco et al., 2020).

As they do in the case of chronic inflammation, in neuroinflammation, sirtuins have mainly an anti-inflammatory effect. However, the literature describes exceptions. For example, inhibition of SIRT2 in mice with accelerated cellular senescence reduced neuroinflammation, as evidenced by reduced glial fibrillar acid protein, IL-1β, IL-6, and TNF-α and increased glutamate receptor subunits GluN2A, GluN2B, and GluA1. However, inhibition of SIRT2 could not reverse cognitive decline or neuroinflammation (Diaz-Perdigon et al., 2020). In this case, SIRT2 demonstrated a temporary pro-inflammatory effect.

Neurodegenerative diseases correlate with aging, as do changes in sirtuin expression (Julien et al., 2009; Jiao, Gong, 2020). It is interesting to note that age-related changes in serum sirtuin can be used as a diagnostic tool (Kumar et al., 2014). For instance, the expression of SIRT1 and SIRT6 is reduced against the background of neurodegenerative diseases (Jiao, Gong, 2020; Pukhalskaia et al., 2020). A high content of SIRT2 is found in Alzheimer’s and Parkinson’s diseases, suggesting that it may contribute to neurodegeneration (Cacabelos et al., 2019).

## Conclusion

Since the discovery of yeast Sir2, studies of sirtuins have focused on their functions in regulating processes associated with aging (Pukhalskaia et al., 2022). Recent research confirms the key role of sirtuins in the pathogenesis of age-related diseases. This makes sirtuins promising targets for research in the field of age-related disease therapy. Indeed, currently underway are many clinical trials aimed at pharmacological modulation of sirtuin activity for the treatment of metabolic, immune, and neurological disorders, as well as cardiovascular and oncological diseases (Curry et al., 2021). Unfortunately, these clinical trials do not always show positive results, which, in all likelihood, may be due to the highly multifaceted functions of sirtuins. However, detailed information about their functions is dynamically accumulated, which hopefully will allow for the implementation of such progressive methods of therapy for age-dependent diseases as soon as reasonably possible

## Conflict of interest

The authors declare no conflict of interest.
